# A Rare Case of Invasive Pneumococcal Disease Presenting With Pneumonia, Meningitis, Septic Arthritis, and Cerebral Infarcts

**DOI:** 10.7759/cureus.99715

**Published:** 2025-12-20

**Authors:** Hassan Waraich, Humayun Haq, Hashir Abdullah

**Affiliations:** 1 Internal Medicine, Manchester University NHS Foundation Trust, Manchester, GBR; 2 Cardiology, Tameside and Glossop Integrated Care NHS Foundation Trust, Ashton-under-Lyne, GBR

**Keywords:** cerebral infarcts, immunocompetent adult, invasive pneumococcal disease, meningitis, pneumococcal pneumonia, septic arthritis

## Abstract

*Streptococcus pneumoniae* (SP) is a common pathogen responsible for a broad spectrum of infections and the leading cause of community-acquired pneumonia; however, invasive and life-threatening disease is rare in immunocompetent individuals. We describe the case of a 60-year-old male who presented with a constellation of neurological and systemic symptoms involving the respiratory, nervous, and musculoskeletal systems, and was subsequently diagnosed with invasive pneumococcal disease (IPD). Despite the severity of his illness, the patient made a remarkable recovery following prompt initiation of aggressive antimicrobial therapy and surgical intervention. This case underscores the importance of early recognition and timely management of IPD, particularly in atypical multisystem presentations occurring in otherwise immunocompetent individuals. To our knowledge, this represents a rare presentation of IPD characterised by the unusual constellation of pneumonia, meningitis, septic arthritis, and cerebral infarcts.

## Introduction

*Streptococcus pneumoniae* (SP) is a gram-positive cocci, which usually colonises the nasopharynx and is transmitted through droplets. It can spread locally within the respiratory tract or spread to distant sites. SP-related diseases are a prominent cause of morbidity and mortality, especially in children and in the elderly population, and are the leading cause of community-acquired pneumonia. In 2005, the World Health Organization estimated that 1.6 million deaths were caused by this organism annually [1]. Its prevalence is largely due to its colonising ability in the nasopharynx; it may be isolated from the nasopharynx of 5-90% of healthy persons, depending on the population and setting, for instance, 5-10% adults without children and 20-60% of school-aged children are carriers [2].

SP manifests in a wide spectrum of clinical syndromes, ranging from non-invasive disorders such as pneumonia, otitis media, rhino-sinusitis, to life-threatening conditions such as sepsis, meningitis, and empyema [1]. Invasive pneumococcal disease (IPD) is characterised by detection of the organism from sterile sites, for instance, cerebrospinal fluid, synovial fluid, or blood [3]. In 2010, the European Centre of Disease Prevention and Control (ECDC) estimated an overall incidence of IPD of 5.2 cases per 100,000 people in European countries. Certain conditions are associated with a greater risk of developing IPD, including chronic lung, kidney, and heart diseases, alcohol abuse, cigarette smoking, and diabetes. Among immunocompromised individuals, additional risk factors include human immunodeficiency virus infection, asplenia, hematological malignancies, organ transplantation, and sickle cell disease. Factors associated with increased mortality in IPD include male sex, age over 45 years, and hospital-acquired infections during the course of the disease [1]. However, these represent only some of the known risk factors and are not an exhaustive list. In our patient, the relevant risk factors were his age, gender, and chronic untreated hypertension.

*Streptococcus pneumoniae* is well documented for causing pneumonia, meningitis, and septic arthritis individually; however, the simultaneous occurrence of these manifestations along with cerebral infarcts is exceptionally rare. IPD should be considered as a differential diagnosis in patients presenting with multisystem involvement, to enable timely initiation of appropriate therapy. 

## Case presentation

A 60-year-old Ghanaian male with a history of untreated hypertension but no other comorbidities or immunosuppressive conditions, presented to the emergency department with a one-day history of sudden-onset confusion, reduced consciousness, expressive dysphasia, neck stiffness, right facial droop, and left-sided weakness. He also reported a painful, swollen right knee. He worked in a bakery, was a lifelong non-smoker, and denied alcohol use or recent travel.

On presentation, his Glasgow Coma Scale (GCS) score was 9/15 (eye opening 2, verbal response 2, motor response 5). Vital signs were stable: heart rate 94 beats per minute, blood pressure 128/68 mmHg, respiratory rate 18/minute, oxygen saturation 96% on room air, and temperature 36.5°C. Physical examination revealed a positive Kernig’s sign and neck stiffness. Cardiovascular and respiratory examinations were unremarkable. Neurologically, he demonstrated expressive aphasia and left-sided hemiparesis. The right knee was erythematous, hot, and tender with a significantly reduced range of motion (ROM).

**Figure 1:**
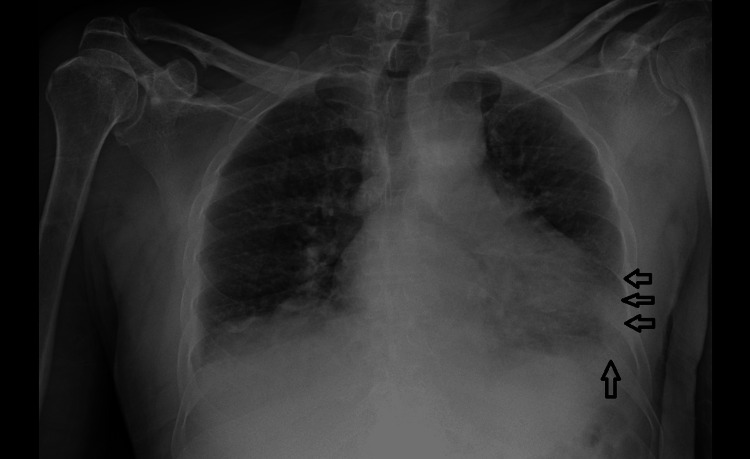
Admission chest X-ray The chest X-ray shows left lower zone consolidation with small bilateral pleural effusions

A chest X-ray revealed left lower zone consolidation with small bilateral pleural effusions consistent with pneumonia and a degree of mild congestion (Figure 1). The presence of neurological symptoms suggestive of a cerebrovascular accident (ischaemic/ haemorrhagic stroke) warranted urgent non-contrast computed tomography (CT) of the head and CT angiography of the head and neck. The CT showed no evidence of acute intracranial hemorrhage, large vessel occlusion, or infarction. Laboratory findings were notable for a C-reactive protein (CRP) of 368 mg/L and a mild leucopenia (Table 1).

**Table 1:** Summary of laboratory and microbiological investigations CRP - C-reactive protein; WCC - white cell count; PLT - platelets; Hb - hemoglobin; RSV - respiratory syncytial virus; SARS-CoV-2 - severe acute respiratory syndrome coronavirus 2; CSF - cerebrospinal fluid; HSV - herpes simplex virus; VZV - varicella zoster virus; PCR - polymerase chain reaction

Test	Result(s)	Reference range/ interpretation
C-reactive protein (CRP) trend	368, 222, 299, 255, 50 mg/L	0-8 mg/L (markedly elevated, trending down)
White cell count (WCC)	3.6 ×10⁹/L	4-10 ×10⁹/L
Platelets	147 ×10⁹/L	150-410 ×10⁹/L (mild thrombocytopenia)
Hemoglobin	123 g/L	130-170 g/L (mild anemia)
Renal and liver function	Within normal limits	-
Human immunodeficiency virus 1/2 and p24 antigen	Not detected	-
Hepatitis B surface antigen / Hepatitis C antibody	Not detected	-
Respiratory viral polymerase chain reaction (PCR) panel (Influenza A/B, RSV, SARS-CoV-2)	Not detected	-
Hemoglobin electrophoresis	Normal adult hemoglobin profile with no abnormal variants	-
Malaria screen and thick film	Negative for Plasmodium parasites and no parasitemia was identified on microscopy	-
Tuberculosis Quantiferon test	Negative	-
Urinary pneumococcal antigen test	Positive	Diagnostic of pneumococcal infection
Blood cultures	*Streptococcus pneumoniae* isolated; sensitive to ceftriaxone, amoxicillin, and clarithromycin; resistant to co-trimoxazole	Confirms pneumococcal bacteremia
Cerebrospinal fluid (CSF) appearance	Clear, straw-colored	-
CSF gram stain	No organisms seen	-
CSF protein	8.67 g/L	0.15-0.45 g/L (markedly elevated)
CSF glucose	<0.6 mmol/L	2.5-4.5 mmol/L (markedly reduced)
CSF white cell count	277 ×10⁶/L (60% neutrophils, 30% lymphocytes)	0-5 ×10⁶/L
CSF red cell count	25 ×10⁶/L	0 ×10⁶/L
CSF PCR	*Streptococcus pneumoniae* DNA detected	Confirms pneumococcal meningitis
CSF viral PCR panel	Negative (HSV-1/2, VZV, enterovirus, parechovirus)	-

Initial differential diagnoses included meningoencephalitis, acute ischaemic stroke, and septic arthritis. Empirical therapy was initiated with intravenous (IV) ceftriaxone 2 g every 12 hours, IV acyclovir 10 mg/kg every eight hours, and IV dexamethasone 10 mg four times daily, in accordance with local hospital policy. 

In accordance with the sepsis protocol, cultures were obtained from all relevant sites, including blood, urine, right knee synovial fluid, and cerebrospinal fluid. Blood and urine cultures were obtained before the initiation of antibiotics, whereas the lumbar puncture and right knee synovial fluid aspiration were performed after empirical therapy had begun. 

Aspirated synovial fluid from the right knee appeared clear with two plus leukocytes but no organisms on gram stain, and cultures were sterile. Within 48 hours, *Streptococcus pneumoniae* was isolated from blood, urine, and cerebrospinal fluid (CSF) samples, susceptible to ceftriaxone.

**Figure 2:**
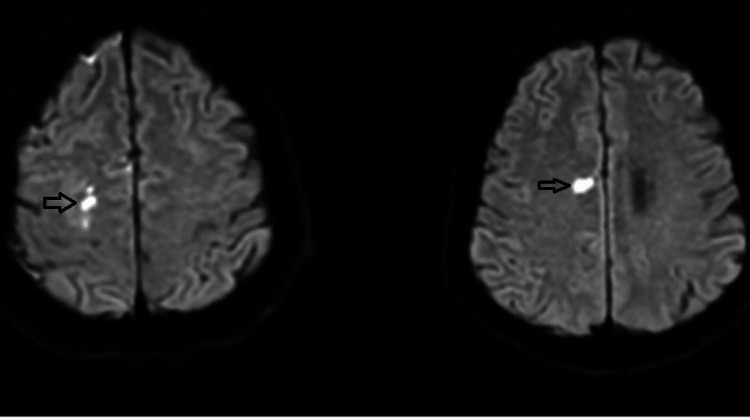
MRI head showing acute infarcts MRI Head non-enhanced study  (axial plane, diffusion-weighted image (DWI), autoAlign technique, deep learning) showing small areas of high frontal cortical and subcortical white matter largest at the right frontal lobe that elicit high signal in DWI suggestive of multiple small acute infarctions.

Magnetic resonance imaging (MRI) of the brain was performed, given the underlying neurological symptoms with a negative CT scan, which demonstrated multiple small acute infarcts, predominantly in the right frontal lobe cortex and subcortical white matter, with additional lesions in the left occipital periventricular region-findings suggestive of embolic infarction (Figure 2). Transthoracic (TTE) and transoesophageal (TOE) echocardiography showed no features of endocarditis with no valvular vegetations or endocardial involvement.

Thus far, the biochemical results were consistent with a severe infection (high CRP), and CSF analysis demonstrated grossly elevated protein (8.67 g/L), low glucose (<0.6 mmol/L), raised white cell count (WCC, 277 ×10⁶/L) with predominantly neutrophils consistent with bacterial meningitis (Table 1). CXR consistent with left side pneumonia and reactive effusion, MRI brain suggestive of embolic infarcts, but no features of endocarditis on TTE and TOE. The right knee aspiration was sterile; however, this was obtained after initiation of antibiotic therapy. SP was isolated in multiple sterile sites, especially blood and CSF, consistent with IPD.

Despite ongoing antibiotic therapy, the patient experienced intermittent pyrexia two weeks into his admission. In view of established cerebral infarctions and bacteremia, there were concerns about evolving intracerebral abscesses, distant deep-seated infection, and endocarditis; therefore, urgent MRI head, CT thorax, abdomen, and pelvis (CT TAP) and repeat echocardiogram were undertaken. Repeat MRI with contrast showed partial resolution of infarcts without abscess formation, CT TAP showed no evidence of deep-seated infection or malignancy, and repeat echocardiography failed to show any features of endocarditis. Serial blood cultures were sterile.

Persistent knee pain with reduced ROM prompted diagnostic arthroscopy, which revealed thick purulent material, hyperaemic and hypertrophic synovium, and inflamed joint surfaces. Synovectomy and debridement were performed. Postoperatively, knee ROM improved from 5-60° preoperatively to 0-90°.

The final diagnosis was invasive pneumococcal disease (IPD) manifesting as pneumonia, meningitis, septic arthritis, and cerebral infarctions. The patient completed a prolonged course of intravenous ceftriaxone, guided by sensitivities. Acyclovir was discontinued after negative viral polymerase chain reaction (PCR), while dexamethasone was continued for a total of four days. Antiplatelet therapy was initiated for secondary stroke prevention.

Following clinical stabilisation and improvement in inflammatory markers, the patient was transferred to a stroke rehabilitation unit. At discharge, his speech had markedly improved, and he had only mild-moderate residual left-sided weakness (Medical Research Council weakness scale grade 3-4/5) but remained functionally independent.

## Discussion

*Streptococcus pneumoniae* remains a major cause of community-acquired infections, but invasive pneumococcal disease (IPD) affecting multiple organ systems is rare, particularly in immunocompetent adults [4]. 

Pneumococcal septic arthritis accounts for 2-4% of all septic arthritis cases in adults and typically affects large joints [5]. It may arise secondary to haematogenous dissemination during bacteraemia. In this case, although the initial synovial aspirate was sterile, arthroscopy confirmed septic arthritis. It is important to note that a negative joint aspirate does not rule out septic arthritis, especially if sampling occurs after the initiation of antibiotics. This underscores the importance of obtaining microbiological samples from all potential sources before starting antimicrobial therapy, as well as maintaining a low threshold for surgical exploration and intervention when clinical suspicion remains high despite negative cultures.

Neurological complications of pneumococcal meningitis, such as ischemic and embolic strokes, are well-recognized sequelae resulting from central nervous system inflammation and vascular involvement. Pathological processes involved include medium-large artery inflammation, cerebral haemorrhage, cerebritis, thrombosis, ventriculitis, and infarction. These complications are associated with one of the highest case-fatality rates among all forms of bacterial meningitis, 22% in adults. The absence of endocarditis on serial echocardiograms (TTE and TOE) is indicative of a localised process resulting in intracerebral infarcts such as meningitis-related vasculitis [6,7].

Although the patient was considered immunocompetent, his age (60 years) warrants consideration of immunosenescence. Advancing age, even without a defined immunosuppressive condition, is associated with reduced immune responsiveness and an increased susceptibility to invasive pneumococcal disease. In addition, his male sex and untreated chronic hypertension further predisposed him to adverse outcomes [1]. The combination of pneumonia, meningitis, septic arthritis, and cerebral infarcts in the absence of endocarditis is exceptionally rare in IPD. This case highlights the versatile manifestations of IPD and the diagnostic challenges it presents, particularly when symptoms span multiple organ systems. Early recognition, timely initiation of targeted antimicrobial therapy, and surgical management of localized infection are critical for reducing morbidity and mortality.

## Conclusions

Invasive pneumococcal disease can present with atypical and multisystem involvement, especially in those with risk factors such as age over 45 years, male gender, cigarette smoking, diabetes, immunosuppression, and cardiovascular disease. Our case illustrates an unusual constellation of pneumococcal pneumonia, meningitis, septic arthritis, and cerebral infarcts occurring simultaneously with the absence of endocarditis. Prompt diagnosis, aggressive antimicrobial therapy, and early surgical intervention were pivotal in achieving recovery. Clinicians should maintain a high index of suspicion for IPD in patients with overlapping neurological, respiratory, and musculoskeletal symptoms, and should aim to obtain microbiological samples before antibiotic administration to maximise diagnostic yield and guide treatment effectively. Following recovery from invasive pneumococcal disease, patients should be counseled regarding immunization against pneumococcus, influenza along with age-appropriate immunizations such as tetanus, diphtheria, pertussis (Tdap) booster, and the recombinant zoster vaccine.
